# The Energy Efficiency Multi-Robot System and Disinfection Service Robot Development in Large-Scale Complex Environment

**DOI:** 10.3390/s23125724

**Published:** 2023-06-19

**Authors:** Chin-Sheng Chen, Feng-Chieh Lin, Chia-Jen Lin

**Affiliations:** 1 Graduate Institute of Automation Technology, National Taipei University of Technology, Taipei 10608, Taiwan; saint@ntut.edu.tw (C.-S.C.); jack12407@ntut.edu.tw (F.-C.L.); 2 Smart Automation Unit, TECO Electric & Machinery Co., Ltd., 10F, No. 3-1, Park St., Nan-Kang, Taipei 11503, Taiwan

**Keywords:** multi-robot system, MRS, energy saving, service robot, VSLAM, EKF

## Abstract

In recent years, multi-robot control systems and service robots equipped with graphical computing have been introduced in various application scenarios. However, the long-term operation of VSLAM calculation leads to reduced energy efficiency of the robot, and accidental localization failure still persists in large-scale fields with dynamic crowds and obstacles. This study proposes an EnergyWise multi-robot system based on ROS that actively determines the activation of VSLAM using real-time fused localization poses by an innovative energy-saving selector algorithm. The service robot is equipped with multiple sensors and utilizes the novel 2-level EKF method and incorporates the UWB global localization mechanism to adapt to complex environments. During the COVID-19 pandemic, three disinfection service robots were deployed to disinfect a large, open, and complex experimental site for 10 days. The results demonstrated that the proposed EnergyWise multi-robot control system successfully achieved a 54% reduction in computing energy consumption during long-term operations while maintaining a localization accuracy of 3 cm.

## 1. Introduction

With the emergence of improved perception technology and computational capabilities, service robots have been upgraded from traditional, simple application fields to large-scale and diverse ones [1]. Recently, robot integration and control have significantly evolved from stand-alone operations to multi-robot-based group activities, where [2] analyze multi-robot motion and task planning, application domain implementation, and improved coordination, and [3] discuss with communication mechanisms, a planning strategy and a decision-making structure. The architecture of the ROS (robot operating system) open-source system [4], combined with the scalability advantages of Linux version upgrades, enables the design of mobile robots and the connection of multi-robot system (MRS) frameworks [5]. ROS offers a collection of robotic software libraries and a reliable navigation mechanism for modular nodes [6], which allows for easy execution and communication and provides a reliable solution for logistics and manufacturing tasks [7]. In comparison to single-robot scenarios, multi-robot field operations occur in more complex and dynamic environments. However, with advancements in SLAM (simultaneous localization and mapping) methods, sensing technology, and multi-sensor fusion algorithms [8], robots are now capable of performing spatial localization and navigation with increased accuracy and efficiency in challenging and dynamic conditions [9]. During the COVID-19 pandemic, multi-robot systems were applied in various areas such as monitoring, food delivery [10], and disinfection [11]. These systems have been instrumental in maintaining social distance while ensuring essential tasks are completed efficiently.

In complex application fields, relying solely on LiDAR for robot localization may result in sparse feature points, potentially causing the robot to lose its sense of direction [12]. Furthermore, the presence of crowds can further aggravate this issue, rendering multi-robot systems unsuitable for large-scale applications due to this limitation. For example, [13] studies the characteristics of laser scanners, and [14] describes SLAM problems and proposes solutions. The fusion of image and LiDAR sensor localization technology can address many of the navigation challenges. Ref. [15] compares and analyzes the mobile robot trajectories calculated by various ROS-based SLAM systems. Ref. [16] proposes to reduce the number of Rao–Blackwellized particle filter (RBPF) particles using adaptive technology. However, the intensive image processing required by visual SLAM (VSLAM), where [17] introduced ORB-SLAM and [18] showed that ORB-SLAM2, may gradually reduce the robot’s energy efficiency over time, even with multi-sensor fusion improving its localization ability [19]. In fields where MRS need to adapt to increasingly complex human-robot collaboration, optimizing robot utilization efficiency will become a critical issue to address in the future. Despite the progress made in MRS, there is currently a lack of an interactive control mechanism that can simultaneously maintain position awareness and optimize energy efficiency. This presents a significant challenge that has yet to be overcome in this field.

This paper proposes an EnergyWise MRS with an interactive regulation mechanism that maintains localization awareness while optimizing energy efficiency. The concept of “leading the way” is introduced, and the switching of VSLAM during the fleet’s navigation process is streamlined, optimized, and built upon the open-source TUW architecture [20]. The MRS presented in this work is capable of adjusting the visual computing resources of each robot based on their relative positions and can be utilized in ROS-based service robots [21]. This research proposes an innovative double-layer Kalman filter algorithm that performs fusion perception calculations for various sensors, including the visual camera, 2D laser scanner, IMUs, and the odometer. To address the cumulative offset error of the odometer and IMU, we propose leveraging ultra-wideband (UWB) [19] technology for global localization, thereby providing a reliable mechanism for error correction. The robot communicates with the MRS in real-time using the energy-saving selector’s calculation mechanism. The MRS utilizes the robot’s localization results as the basis for decision-making to initiate visual computation, conserving computational resources.

This study deployed three disinfection service robots in a complex and open indoor space with moving crowds to evaluate MRS design performance and its energy-saving capability. The experiment lasted for 100 h and ensured no localization failure problem. During the verification process, the leader robot initiates VSLAM calculations and performs multi-dimensional visual space perception and obstacle detection in the field. It then sends the real-time fused poses back to the MRS for processing. In evaluating power consumption statistics and comparing localization methods by each sensor, the MRS uses relative position deviations on robots to control the on-off switching of the VSLAM calculation, thereby accomplishing the energy-saving objective.

The contributions can be summarized as follows.

- The novel EnergyWise MRS and service robot system based on the ROS and TUW frameworks have been introduced. It accelerates development speed and ensures software scalability.
- By utilizing the 2-level multi-sensor fusion EKF algorithm and global UWB technology integrated into both the MRS and robots, localization precision is enhanced to 3 cm.
- The innovative energy-saving selector algorithm improves VSLAM calculation regulation, resulting in a 50% reduction in energy consumption. This mechanism offers a power-efficient solution for MRS research.

## 2. Related Works

### 2.1. The ROS-Based Multi-Robot System

Since the inception of multi-robotics research in the 1980s, this field has experienced substantial growth. It is easier to perform tasks efficiently and cost-effectively with MRS than with single-robot systems due to their scalability, reliability, flexibility, and versatility [22]. MRS research spans multiple domains, allowing robots to collaborate and support each other to accomplish interactive tasks [23]. An example of group control in MRS is the classic Swarm robotic system, which set the precedent for research in this field [24]. MRS has facilitated the development of industrial and service robots by connecting individual robots into a coordinated system, allowing for valuable improvements in many processes. MRS can enhance greater accuracy, quality, and cost savings by executing routine service procedures [25].

With the trend towards open-source systems, ROS-based MRS have gradually developed. Open-source tools such as Player/Stage [26] and Gazebo [27] have been developed to support system simulation for multi-robot control. In the development of MRS, many challenging issues related to distributing computing, collaboration, and coordination must be addressed. Therefore, the development and deployment of MRS applications are challenging tasks, as real-time integration of robot modules and services is a crucial consideration. To simplify the development of multi-robot applications, researchers have proposed various middleware frameworks [28] that provide programming abstractions to manage hardware and application complexity and heterogeneity [29]. Currently, a master-slave network node architecture can be utilized to enable multi-robot communication via message passing using multi-master [30]. Task allocation can be effectively combined with open-source solutions. For example, TUW can aid in multi-robot tasks and path planning by designing routes on a search graph for multiple robots. This approach involves constructing a search graph from a pixel map and utilizing an extended priority planning method to locate paths for multiple robots [31]. In ROS-based frameworks, navigation and control commands for robots can be designed and executed within a global map, enabling MRS to plan and execute tasks efficiently [32].

### 2.2. Multi-Sensor Fusion Localization Technology in MRS

In large-scale environments, various environmental characteristics, such as glass that cannot be sensed by LiDAR or moving people [33], can cause environmental variations, which may result in the failure of robot localization. To address this challenge, fusion algorithms can be utilized to process and analyze the information collected by sensors. This enables the robot system to make informed decisions about the end application, improve localization accuracy, and avoid the problem of insufficient information provided by a single sensor [34]. The Kalman filter (KF), a well-known autoregressive filtering algorithm for linear systems, is commonly used for multi-sensor data fusion. This algorithm was first published in 1960 by Rudolf Emil Kálmán [35]. The KF algorithm compares the predicted model solution to the actual data obtained from observations. By considering the state distribution of each data and solution over time, the KF algorithm finds the optimal solution for the state over time. Due to its wide applicability, it is utilized in various fields and has undergone many modifications to compensate for its limitation of only providing optimal solutions in linear systems, such as the lossless Kalman filter (unscented Kalman filter, UKF) [36], EKF, among others.

The extended Kalman filter (EKF) is commonly used for state estimation and data fusion in robot navigation. In the MRS research field, multi-sensor perception techniques are employed for robot localization, spatial exploration, and cognition. This enables multiple robots to achieve greater environmental sensor coverage and improve the accuracy of object location estimation [37]. Additionally, the integration of target position estimation techniques enables robots to re-establish contact after extended periods of time and proposes a fully distributed team planning algorithm that utilizes limited shared information when available [38]. This article utilizes the EKF for multi-robot perception fusion localization applications, based on recent developments in speed estimation and obstacle avoidance for robots [39], thus inheriting the aforementioned advantages.

Due to the complexity of indoor environments, research on UWB indoor localization has become increasingly widespread [40]. In addition to fulfilling the needs of indoor industrial localization [41], it is also used in indoor MRS applications [42]. Its high accuracy makes it particularly suitable for supporting more extensive collaboration and multi-robot application scenarios compared to other wireless localization solutions [43]. UWB technology has become increasingly commercialized and offers advantages such as high time resolution, large transmission capacity, and relatively low power consumption. Due to these benefits, UWB is being used as an alternative to more complex and expensive motion capture systems. By combining UWB anchors and tags, reference coordinate information can be obtained for global localization, making it a suitable choice for indoor localization applications [42]. Relevant research confirms that UWB indoor localization accuracy can approach 0.5 m, making it suitable for use in service robots and in MRS for global robot localization and configuration [43]. 

### 2.3. Power Efficiency with Service Robot and MRS Requirement

Currently, energy-saving designs have been developed and implemented in the field of service robots; the objective is to minimize energy waste while enhancing operational efficiency [44]. Additionally, the COVID-19 pandemic has brought about an urgent need for contactless services to maintain social distancing [9], and the service efficiency of robots is increasingly being emphasized [45]. In terms of the design and selection of service robots, there is a growing focus on lightweight materials and streamlined appearances to reduce the additional weight on the carrier. For control systems, high-energy efficient drive and control components are chosen, along with the incorporation of energy recovery units. 

Discussions on energy-saving measures related to ROS open-source architecture and MRS have also been explored in the academic field. For instance, there have been studies on path planning efficiency that aim to enhance the robot’s mobility and power usage and also focus on the optimization of arrangements to reduce the robot’s repeated detours [46]. While localization accuracy improved with graphical processing, power consumption also increased. As a result, energy-saving research on VSLAM has been conducted in recent years. This includes using FPGA-embedded system designs as the basis for basic VSLAM calculations [47], analyzing requirements based on dataset efficiency [48], and selecting related visual algorithms. For the most energy-efficient application, ORB-SLAM2 will be selected, where [49] mobile robots run on the NVidia Jetson platform, enabling high performance while maintaining low power consumption. Ref. [50] studies the use of visual SLAM methods in autonomous robots and their effect on power consumption.

The summary of the aforementioned studies includes two aspects: utilizing high-performance computing hardware to enhance VSLAM performance and analyzing the computational efficiency of various VSLAM algorithms to select the most efficient one. No matter which method is employed, the most efficient approach is to reduce the utilization of VSLAM. Based on this concept, this paper proposes an energy-saving selector method aimed at improving the energy efficiency of MRS.

## 3. The Control Theory of EnergyWise Multi-Robot System

This section provides the control theory of the energy-saving selector in the MRS framework. Firstly, it describes the MRS and UWB anchor architectures in detail, followed by the introduction of a service robot developed using ROS. The control process of the energy-saving selector in the MRS is then explained, emphasizing its role in improving power efficiency for each robot. Finally, the 2-level multi-sensor EKF and the procedure for localization correction with UWB technology are presented in detail.

### 3.1. EnergyWise Multi-Robot System (MRS) System Architecture

#### 3.1.1. Control Architecture

**Figure 1:**
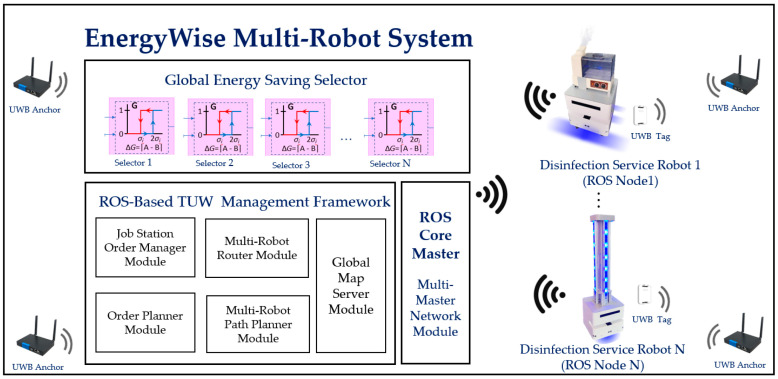
EnergyWise multi-robot system and ROS-based service robot control architecture.

The EnergyWise MRS architecture is based on the open-source ROS framework, as shown in Figure 1. The system consists of an energy-saving selector, TUW management framework, and core master. Control algorithms are used for active switching of VSLAM and robot navigation, task assignment, and network communication. The service robot adopts a wheeled mobile platform and disinfection module. MRS and robot control commands are transmitted via Wi-Fi using ROS node topics. UWB anchors are placed around the workspace, with a transmission range of 30 m, to provide global reference coordinates transmitted by UWB tags mounted on each robot.

In the TUW framework, the job station order manager module and the order planner module are used to set up reference sites on the map, define tasks, avoid path conflicts, and allow tasks to be assigned. The multi-robot router and path planner module synchronizes task settings with the global map and creates navigation path routing, connecting with real-time coordinates. The core master of multi-robot communication employs the ROS topic/message communication format as a mechanism for command and information transmission between robots and MRS.

The leading robot functions in conjunction with the EnergyWise MRS to achieve the energy-saving objective. Referring to Figure 1, the first robot, Robot(1), is defined as the leader robot, Robot(2) is the second robot in the order, and so on up to Robot(N). All robots follow behind Robot(1) according to the pre-defined path for disinfection tasks. The leader robot, Robot(1), simultaneously activates the 2D LiDAR and depth camera to detect changes in the environment. In contrast, the following robots, Robot(2) to Robot(N), deactivate the depth camera to save energy. When Robot(1) detects an increase in obstacles in the surrounding environment, the multi-robot controller notifies the subsequent robots to activate the VSLAM to enhance localization and obstacle avoidance capabilities. 

#### 3.1.2. The ROS Based Disinfection Service Robot

**Figure 2:**
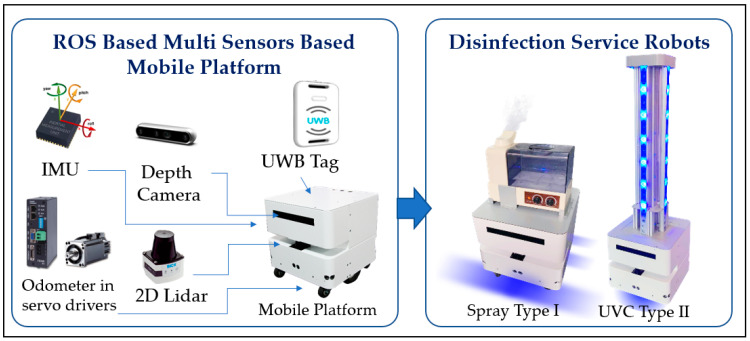
The ROS-based multi-sensors mobile platform and design of disinfection service robots.

As illustrated in Figure 2, the service robot incorporates several sensing devices, including a laser rangefinder, a depth camera, odometry via servo drives, an IMU, and a UWB tag. The odometer utilizes encoder feedback from the servo drives, while the UWB tag transmits the robot’s global localization reference coordinates in two dimensions. The mobile platform can house a sprayer and UVC disinfection device, which are designed as a type I spraying robot and a type II UVC disinfection service robot, respectively. It is possible, however, to solve most spatial localization problems with 2D SLAM fusion with a visual calculation algorithm in the localization function. However, the visual camera combined with the localization calculation process consumes around 25 W of power. As the robot depends on batteries for calculation and power supply, and energy consumption is fixed in the dynamic system, minimizing computational consumption during navigation is the primary focus of the issue.

**Figure 3:**
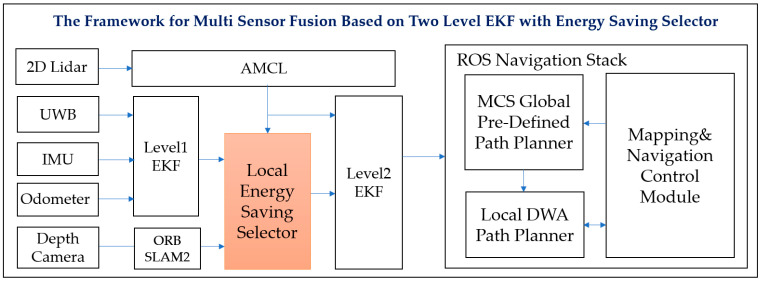
The framework for multi-sensor fusion based on 2-level EKF with energy-saving selector.

The energy-saving selector proposed in this paper was implemented by using the 2-level EKF architecture with multiple sensors, as shown in Figure 3. The design aims to enhance the robot’s energy efficiency during navigation by utilizing a double-layer EKF fusion and the energy-saving selector. In this design, level 1 EKF fusion is executed using IMU, odometer, and global UWB to provide the robot’s global localization coordinates. Then, the resulting output is switched with 2D and visual localization to the energy-saving selector to determine whether to activate the visual calculation process and conduct level 2 perceptual fusion. The final localization information is used as the coordinates and localization reference for the robot to perform tasks in the navigation stack. 

The MCS global planner enables the robot to set its departure and destination goals using a pre-defined path and transmits motion trajectories that the local planner can reference. The DWA algorithm is employed to calculate the robot’s optimal speed and angle of movement. In conjunction with the map information from the mapping and navigation control module, it generates paths and achieves obstacle avoidance. In addition to meeting the MRS task configuration requirements, this design also features a multi-sensor design. The energy-saving selector is used to select and perform visual calculations, achieving energy-saving effects. The architecture and operating mechanism of the energy-saving selector will be introduced in detail in Section 3.2, and the calculation design of 2-level multi-sensor fusion will be discussed in Section 3.3.

### 3.2. The Global Energy-Saving Selector

**Figure 4:**
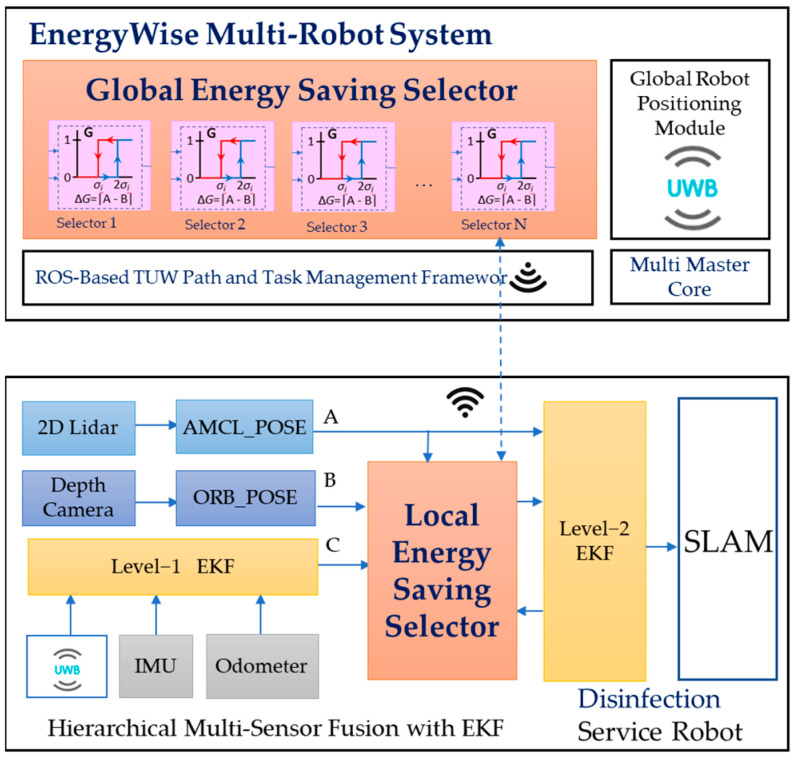
The 2−level EKF and energy-saving selector in EnergyWise MRS.

The EnergyWise multi-robot system, as shown in Figure 4, consists of a local energy-saving selector within each individual robot and a global energy-saving selector in the multi-robot control system. By reducing the computation of VSLAM, the MRS achieves optimal energy-saving effects. The selector selects “Pose B” and “Pose C” according to the deviation between “Pose A, B, and C”. Among them, “Pose A” represents the localization information calculated by 2D LiDAR through AMCL. “Pose B” corresponds to the localization information computed by the depth camera using ORB SLAM2. “Pose C” refers to the localization information obtained by blending three sources: UWB, IMU, and odometer, through level 1 EKF fusion. The UWB localization information is provided by MRS, while the IMU and odometer localization information is obtained through calculations using motor encoders on the robot. Finally, the 2-level EKF fusion calculates the final pose information of the robot based on either “Pose A” or the energy-saving selector’s choice of “Pose B” or “Pose C”.

**Figure 5:**
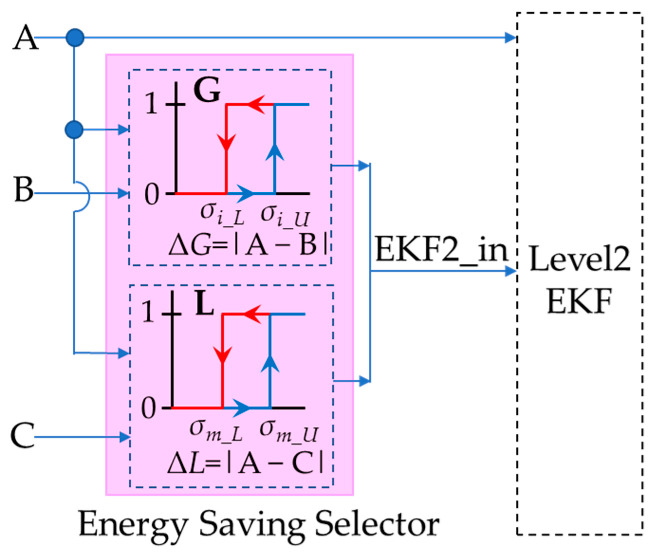
Design structure of energy−saving selector.

The energy-saving selector consists of two hysteresis selectors, as shown in Figure 5. Hysteresis switching architecture prevents the selector from switching too quickly, which could result in instability in robot operation, and selector G is located within MRS and associated with Equation (1).

Δ*G*(k) = |*A*(k)−*B*(k)| Δ*G*(k) > *σ_i_U_* → EKF2_in(k) = *B*(k) Δ*G*(k) < *σ_i_L_* → EKF2_in(k) = *C*(k) (1)

Δ*G* represents the deviation between Pose A and Pose B, while *σ_i_* represents a variance that can be set according to environmental features. When Δ*G* is greater than *σ_i_U_*, level 2 EKF uses Pose B as input, and when Δ*G* is less than *σ_i_L_*, level 2 EKF uses Pose C as input, respectively. Selector L is situated in the service robot system and is represented by Equation (2).

Δ*L*(k) = |*A*(k)−*C*(k)| Δ*L*(k) > *σ_m_U_* → EKF2_in(k) = *B*(k) Δ*L*(k) < *σ_m_L_* → EKF2_in(k) = *C*(k) (2)

Δ*L* represents the deviation between Pose A and Pose C, while *σ_m_* represents a variance that can be adjusted based on the characteristics of the robot and environmental features. When Δ*L* exceeds *σ_m_U_*, level 2 EKF uses Pose B as input; conversely, when Δ*L* is less than *σ_m_L_*, level 2 EKF uses Pose C, and selector L is in the individual service robot and expressible by Equation (2).

**Figure 6:**
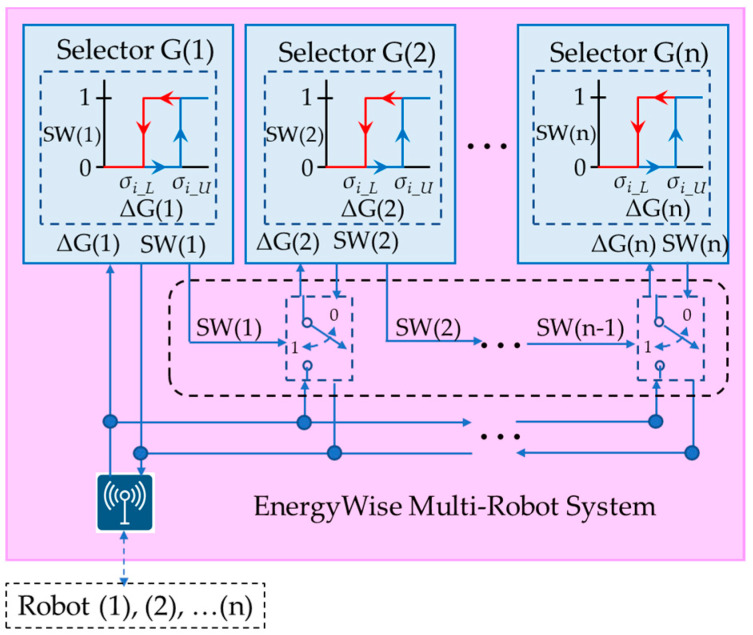
Energy-saving selector switching architecture in EnergyWise MRS.

When the system starts, the first leading robot turns on the visual perception device to detect the dynamic characteristics of the environment. It then transfers the results to the MRS system, which determines the switching timing for VSLAM calculations for other robots. Figure 6 shows the energy-saving selector switching architecture on the MRS side. 

During the operation of the MRS, the system obtains the deviation ΔG(1) of Robot(1) and evaluates it using selector G(1) in Figure 6. Subsequently, the system generates and outputs SW(1), which is then sent back to Robot(1) through a wireless communication topic in selector L. In this condition, SW(1) serves as the basis for deciding whether to activate selector G(2). Following the design logic, the activation of selector G(3) is determined based on SW(2), and similarly, the activation of selector G(n) is decided based on SW(n−1).

**Figure 7:**
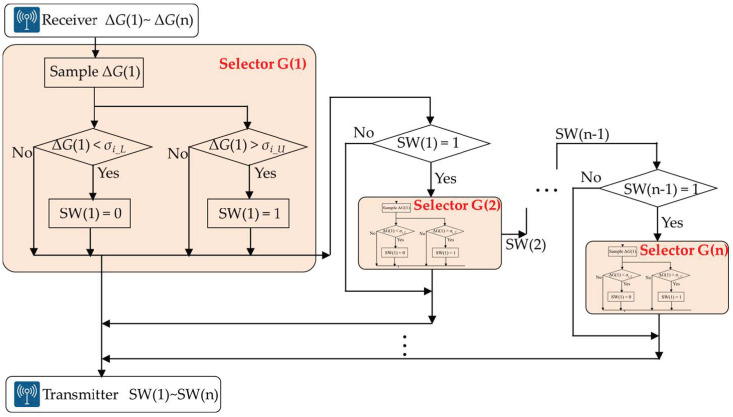
The control flow chart of the energy-saving selector on the EnergyWise multi-robot system.

The control flow diagram of the energy-saving selector on EnergyWise MRS is shown in Figure 7. According to the hysteresis switching rule in Equation (1), the system obtains the deviation ΔG(1) of Robot(1) and sends ΔG(1) to selector G(1). If ΔG(1) > *σ_i_U_*, SW(1) is set to 1; conversely, if ΔG(1) < *σ_i_L_*, SW(1) is set to 0. MRS sends SW(1) back to Robot(1), and the result of SW(1) also serves as the activation criterion for selector G(2). It is assumed that selector G(3) is activated by SW(2), likewise, selector G(n) is activated by SW(n−1). As long as the pose deviation ΔG(k) of Robot(k) remains below *σ_i_U_*, Robots (k + 1~n) can deactivate the depth camera and ORB-SLAM2 to save power, ultimately achieving energy efficiency.

**Figure 8:**
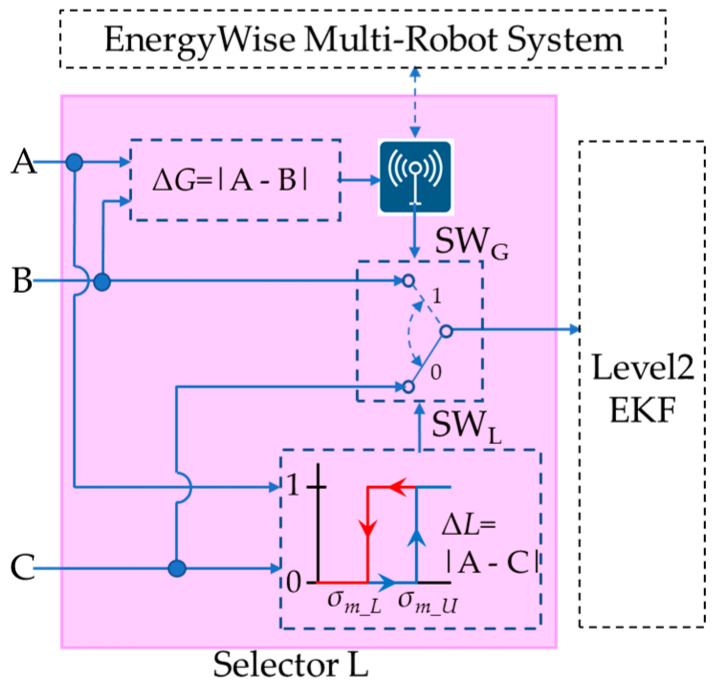
Selector L switching architecture of service robot.

Figure 8 illustrates the energy selector L architecture designed for service robots. Equation (1) is employed to calculate Δ*G*, which is then transmitted to the MRS via an ROS topic. The MRS provides the SW_G_ switching signal to the robot, allowing it to determine the pose between Pose B and Pose C. However, environmental factors can potentially impact Pose C, leading to offset issues. To address this, the service robot computes Δ*L* using Equation (2) and utilizes SW_L_ as input for the level 2 EKF when dealing with Pose B and Pose C.

**Figure 9:**
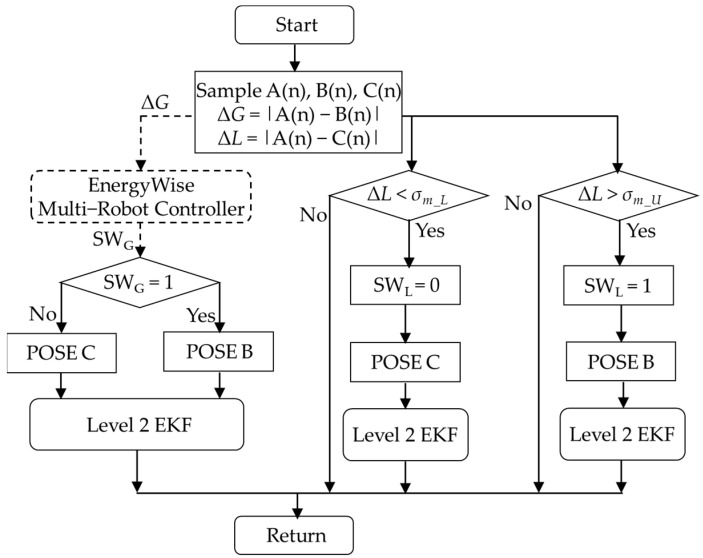
Selector L switching flow chart in service robot.

The control flow chart of selector L in the service robot system is illustrated in Figure 9. In this process, the robot samples Pose A, B, and C. On the left side, Δ*G* is calculated according to Equation (1) and transmitted to the MRS. The MRS responds with the SW_G_ signal to determine the selection of Pose B and Pose C. On the right side, the Δ*L* signal is obtained by utilizing the hysteresis switching rule defined in Equation (2). The process inputs selector L. When Δ*L* is greater than *σ_m_U_*, the output is SW_L_ = 1, and when Δ*L* is less than *σ_m_L_*, the output is SW_L_ = 0. In addition to waiting for commands from the MRS, this method also enables the robot to make independent judgments. This enables an energy-saving synchronous control approach while reducing VSLAM computing resources, ultimately enhancing system efficiency. 

### 3.3. 2-Level Multi-Sensor Fusion EKF

The MRS design combines local and global localization and introduces a 2-level multi-sensor fusion EKF computing architecture, as shown in Figure 4. The pose calculation results for each level are used as the basis for the decision-making process for image localization resources. With the design of hierarchical multi-sensor fusion and the EKF method, the aim is to determine the pose and velocity of a mobile robot over time. As a nonlinear dynamic system, it can be characterized and defined as in Equation (3).

(3) $$x_{k}=f\left(x_{k-1}\right)+w_{k-1}$$

where $x_{k}$ is the robot’s system state at time k, f is a nonlinear state transition function, and $w_{k-1}$ is the gaussian noise of the system process, which is assumed to be normally distributed.

The five-dimensional state vector, $x_{k}$ involves the robot’s pose, orientation, and respective velocities. $l_{x,k}$ is the x-direction position status of the robot, $l_{y,k}$ is the y-direction position status of the robot, and $\theta_{k}$ is the angular status of the robot, $v_{k-1}$ and ${\dot{\theta }}_{k-1}$ are the line speed commands given to the robot, respectively. And the angular velocity command can be defined as in Equation (4).

(4) $$x_{k}={\left(l_{x,k}l_{y,k}\theta_{k}v_{k}{\dot{\theta }}_{k}\right)}^{T}$$

$f(x_{k-1})$ is the nonlinear system transfer function of the robot motion model, as shown in Equation (5).

(5) $$f(x_{k-1})=\left[\begin{array}{c} l_{x,k-1}\\ l_{y,k-1}\\ \theta_{k-1}\\ v_{k-1}\\ {\dot{\theta }}_{k-1} \end{array}\right]=\left[\begin{array}{c} v_{k-1}*\cos\left(\theta_{k-1}\right)*∆t\\ v_{k-1}*\sin\left(\theta_{k-1}\right)*∆t\\ {\dot{\theta }}_{k-1}*∆t\\ v_{k-1}\\ {\dot{\theta }}_{k-1} \end{array}\right]=\left[\begin{array}{c} f1\\ f2\\ f3\\ f4\\ f5 \end{array}\right]$$

Euler angles represent rotational values. Furthermore, the observation function of the sensor can be defined as in Equation (6).

(6) $$z_{k}=h\left(x_{k}\right)+v_{k}$$

$z_{k}$ is the k item of the robot pose state measured at time t. The robot pose state $z_{k}$ measured by the sensor used by the robot can be defined as the following Equation (7), where $v_{k}$ is the Gaussian noise of a sensor state measurement and $h\left(x_{k}\right)$ is the system model for converting sensor data into the robot state after sensor measurement.

(7) $$z_{k}={\left(z_{l_{x,k}}z_{l_{y,k}}z_{\theta_{k}}z_{v_{k}}z_{{\dot{\theta }}_{k}}\right)}^{T}$$

It is the first step in the algorithm to perform a prediction step that projects the current state estimation and error covariance forward in time. Equations (8) and (9) represent this process.

(8) $${\hat{x}}_{k}=f\left(x_{k-1}\right)$$

(9) $${\hat{P}}_{k}=FP_{k-1}F^{T}+Q$$

𝑓 is a standard kinematic model based on Newtonian mechanics and used in robot motion. ${\hat{x}}_{k}$ refers to the estimation of the robot state at time t item k. Furthermore, since EKF linearizes the nonlinear system, the physical meaning of F is the Jacobian matrix after linearizing f, as shown in Equation (10).

(10) $$F=\left[\begin{array}{ccccc} 1 & 0 & {-v}_{k-1}*\sin\left(\theta_{k-1}\right)*∆t & \cos\left(\theta_{k-1}\right)*∆t & 0\\ 0 & 1 & v_{k-1}*\cos\left(\theta_{k-1}\right)*∆t & \sin\left(\theta_{k-1}\right)*∆t & 0\\ 0 & 0 & 1 & 0\;\;∆t & \\ 0 & 0 & 0 & 1\;\;0 & \\ 0 & 0 & 0 & 0\;\;1 & \end{array}\right]$$

The estimated error covariance, *P*, is then perturbed by *Q*, the process noise covariance. ${\hat{P}}_{k}$ is the state covariance matrix estimated by EKF, as shown in (11). Q is the covariance matrix of state noise, as shown in Equation (12). As each state is measured by its corresponding sensor, a matrix describes the covariance of all state measurement noises.

(11) $${\hat{P}}_{k}=\left[\begin{array}{c} \begin{array}{c} \begin{array}{cccc} {\hat{\sigma }}_{l_{x}|k-1}^{2} & {\hat{\sigma }}_{l_{x}l_{y}|k-1} & {\hat{\sigma }}_{l_{x}\theta |k-1} & \begin{array}{cc} {\hat{\sigma }}_{l_{x}v|k-1} & {\hat{\sigma }}_{l_{x}\dot{\theta }|k-1} \end{array}\\ {\hat{\sigma }}_{l_{y}l_{x}|k-1} & {\hat{\sigma }}_{l_{y}|k-1}^{2} & {\hat{\sigma }}_{l_{y}\theta |k-1} & \begin{array}{cc} {\hat{\sigma }}_{l_{y}v|k-1} & {\hat{\sigma }}_{l_{y}\dot{\theta }|k-1} \end{array}\\ {\hat{\sigma }}_{\theta l_{x}|k-1} & {\hat{\sigma }}_{\theta l_{y}|k-1} & {\hat{\sigma }}_{\theta |k-1}^{2} & \begin{array}{cc} {\hat{\sigma }}_{\theta v|k-1} & {\hat{\sigma }}_{\theta \dot{\theta }|k-1} \end{array}\\ \begin{array}{c} {\hat{\sigma }}_{vl_{x}|k-1}\\ {\hat{\sigma }}_{\dot{\theta }l_{x}|k-1} \end{array} & \begin{array}{c} {\hat{\sigma }}_{vl_{y}|k-1}\\ {\hat{\sigma }}_{\dot{\theta }l_{y}|k-1} \end{array} & \begin{array}{c} {\hat{\sigma }}_{v\theta |k-1}\\ {\hat{\sigma }}_{\dot{\theta }\theta |k-1} \end{array} & \begin{array}{cc} \begin{array}{c} {\hat{\sigma }}_{v|k-1}^{2}\\ {\hat{\sigma }}_{\dot{\theta }v|k-1} \end{array} & \begin{array}{c} {\hat{\sigma }}_{v\dot{\theta }|k-1}\\ {\hat{\sigma }}_{\dot{\theta }|k-1}^{2} \end{array} \end{array} \end{array} \end{array} \end{array}\right]$$

(12) $$Q=\left[\begin{array}{c} \begin{array}{c} \begin{array}{cccc} \sigma_{Q,l_{x,k-1}}^{2} & 0 & 0 & \begin{array}{cc} 0 & 0 \end{array}\\ 0 & \sigma_{Q,l_{y,k-1}}^{2} & 0 & \begin{array}{cc} 0 & 0 \end{array}\\ 0 & 0 & \sigma_{Q,\theta_{k-1}}^{2} & \begin{array}{cc} 0 & 0 \end{array}\\ \begin{array}{c} 0\\ 0 \end{array} & \begin{array}{c} 0\\ 0 \end{array} & \begin{array}{c} 0\\ 0 \end{array} & \begin{array}{cc} \begin{array}{c} \sigma_{Q,v_{k-1}}^{2}\\ 0 \end{array} & \begin{array}{c} 0\\ \sigma_{Q,{\dot{\theta }}_{k-1}}^{2} \end{array} \end{array} \end{array} \end{array} \end{array}\right]$$

In the next step of the algorithm, we perform a correction step, which is summarized in the equations below.

(13) $$K={\hat{P}}_{k}H^{T}(H{\hat{P}}_{k}H^{T}+R{)}^{-1}$$

(14) $$x_{k}={\hat{x}}_{k}+K=K(z-H{\hat{x}}_{k})$$

(15) $$P_{k}=(I-KH){\hat{P}}_{k}(I-KH{)}^{T}+KRK^{T}$$

The Kalman gain can be calculated using the measurement covariances, *R* and *P*_𝑘_, as well as the observation matrix, *H*. $P_{k}$ is the covariance matrix between the updated robot estimated state $x_{k}$ and the state estimate, as shown in (17), and R is the covariance matrix of the sensor noise and can be defined as in Equation (18). The gain is utilized to update the state vector and covariance matrix. Besides, the Joseph form covariance is applied to update Equation (18) to promote filter stability by ensuring that *P*_𝑘_ remains positive and semi-definite.

(16) $$K_{k}=\left[\begin{array}{c} \begin{array}{c} \begin{array}{cccc} K_{l_{x}|k} & 0 & 0 & \begin{array}{cc} 0 & 0 \end{array}\\ 0 & K_{l_{y}|k} & 0 & \begin{array}{cc} 0 & 0 \end{array}\\ 0 & 0 & K_{\theta |k} & \begin{array}{cc} 0 & 0 \end{array}\\ \begin{array}{c} 0\\ 0 \end{array} & \begin{array}{c} 0\\ 0 \end{array} & \begin{array}{c} 0\\ 0 \end{array} & \begin{array}{cc} \begin{array}{c} K_{v|k}\\ 0 \end{array} & \begin{array}{c} 0\\ K_{\dot{\theta }|k} \end{array} \end{array} \end{array} \end{array} \end{array}\right]$$

(17) $$P_{k}=\left[\begin{array}{c} \begin{array}{c} \begin{array}{cccc} \sigma_{l_{x}|k}^{2} & \sigma_{l_{x}l_{y}|k} & \sigma_{l_{x}\theta |k} & \begin{array}{cc} \sigma_{l_{x}v|k} & \sigma_{l_{x}\dot{\theta }|k} \end{array}\\ \sigma_{l_{y}l_{x}|k} & \sigma_{l_{y}|k}^{2} & \sigma_{l_{y}\theta |k} & \begin{array}{cc} \sigma_{l_{y}v|k} & \sigma_{l_{y}\dot{\theta }|k} \end{array}\\ \sigma_{\theta l_{x}|k} & \sigma_{\theta l_{y}|k} & \sigma_{\theta |k}^{2} & \begin{array}{cc} \sigma_{\theta v|k} & \sigma_{\theta \dot{\theta }|k} \end{array}\\ \begin{array}{c} \sigma_{vl_{x}|k}\\ \sigma_{\dot{\theta }l_{x}|k} \end{array} & \begin{array}{c} \sigma_{vl_{y}|k}\\ \sigma_{\dot{\theta }l_{y}|k} \end{array} & \begin{array}{c} \sigma_{v\theta |k}\\ \sigma_{\dot{\theta }\theta |k} \end{array} & \begin{array}{cc} \begin{array}{c} \sigma_{v|k}^{2}\\ \sigma_{\dot{\theta }v|k} \end{array} & \begin{array}{c} \sigma_{v\dot{\theta }|k}\\ \sigma_{\dot{\theta }|k}^{2} \end{array} \end{array} \end{array} \end{array} \end{array}\right]$$

(18) $$R=\left[\begin{array}{c} \begin{array}{c} \begin{array}{cccc} \sigma_{R,l_{x,k-1}}^{2} & 0 & 0 & \begin{array}{cc} 0 & 0 \end{array}\\ 0 & \sigma_{R,l_{y,k-1}}^{2} & 0 & \begin{array}{cc} 0 & 0 \end{array}\\ 0 & 0 & \sigma_{R,\theta_{k-1}}^{2} & \begin{array}{cc} 0 & 0 \end{array}\\ \begin{array}{c} 0\\ 0 \end{array} & \begin{array}{c} 0\\ 0 \end{array} & \begin{array}{c} 0\\ 0 \end{array} & \begin{array}{cc} \begin{array}{c} \sigma_{R,v_{k-1}}^{2}\\ 0 \end{array} & \begin{array}{c} 0\\ \sigma_{R,{\dot{\theta }}_{k-1}}^{2} \end{array} \end{array} \end{array} \end{array} \end{array}\right]$$

Updates are made to the state vector and covariance matrix using the gain. As part of updating Equation (11), Joseph form covariance is applied to promote filter stability by ensuring that *P*_𝑘_ remains positive and semi-definite. As specified in the standard EKF formulation, H represents the Jacobian matrix of the observation model function H. In the allowed state estimation matrix, H represents the transition matrix for sensor state measurement. As indicated by the matrix element H, a state is present among the estimated variables, which will be discussed in the subsequent section on simulation.

## 4. MRS Experimental Result in Large Scale Complex Field

To validate the energy-saving effectiveness of EnergyWise MRS, this section conducted experiments with three robots in four different scenarios. Additionally, it explains the localization performance of the 2-level EKF in large-scale, complex environments.

### 4.1. The Experimental Field and Testing Scenario

**Figure 10:**
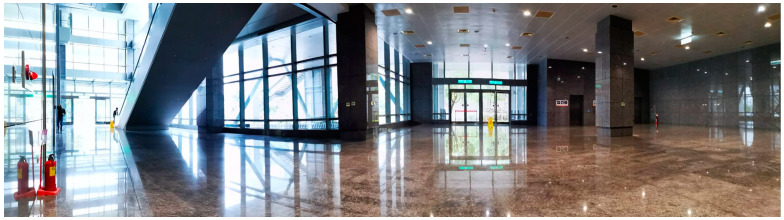
Panoramic overview of the experimental site (size is about 16 m × 15 m).

The experiment aimed to utilize the proposed EnergyWise MRS to enhance the energy-saving efficiency of VSLAM calculations in the robot system and test it in an office building during the COVID-19 pandemic. It involved three disinfection service robots, one equipped with a UVC sterilization lamp, and the other two equipped with disinfectant sprayers. Over 10 working days, the experiment spanned 100 h. As shown in Figure 10, the test site has complex and open environmental characteristics, including issues of light penetration and reflection from the ground and walls, as well as occasional dynamic pedestrian obstacles.

**Figure 11:**
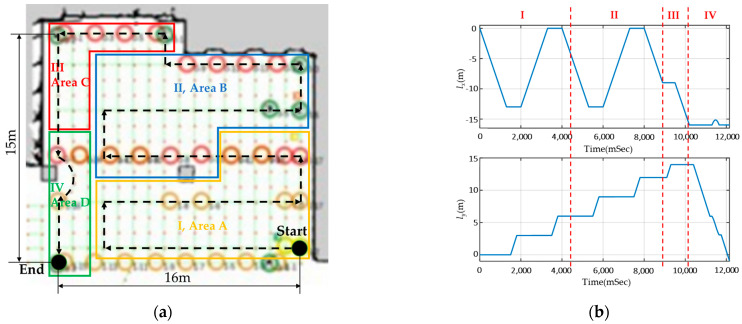
The situational block of the experimental field and the time domain diagram of the motion path: (**a**) The field has an area of 16 m × 15 m and is divided into four scenarios: I, II, III, and IV. (**b**) The time−domain diagram illustrates the motion path over time, with the horizontal axis representing time in seconds and the vertical axis representing the path in meters; the path is also segmented into scenarios I, II, III, and IV.

The testing ground measures approximately 16 m × 15 m, with a distance of 1 m between each grid point, as shown in Figure 11a. The navigation process can be divided into four scenarios: I, II, III, and IV, with the disinfection path planned as a black dotted line. According to Figure 11b, the robot travels at an approximate velocity of 1 m/s, taking around 130 s to travel from the starting point to the endpoint, with movement along the *x* and *y* axes, respectively.

**Figure 12:**
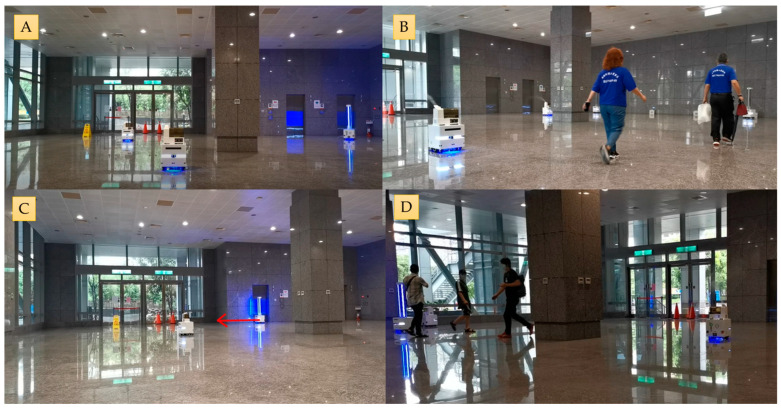
Multi-disinfection service robots operating in four different scenarios in the real world: (**A**) Scenario I: Operating in area A without any obstacles. (**B**) Scenario II: Disinfection is carried out in area B, where fast-moving crowds may appear as obstacles. (**C**) Scenario III: Disinfection is carried out in area C, which has environmental variations. (**D**) Scenario IV: During the operation in area D, crowds of onlookers appeared and interfered with the robot’s progress.

As shown in Figure 12, in scenario I, there is no obstacle interference, and the SLAM task can be accomplished using only 2D LiDAR. As in scenario II, there is a spontaneous crowd that does not interfere with the disinfection task, and the robot follows its predetermined path. In scenario III, despite minor environmental deviations, stable localization performance is achieved with UWB and 2D LiDAR. Finally, in scenario IV, sudden crowd interference disrupts the robot’s operation, but with VSLAM, the robots can still complete the task.

### 4.2. The 2-Level EKF Experiment Result 

#### 4.2.1. The Sensors Performed in the Experiment Field

**Figure 13:**
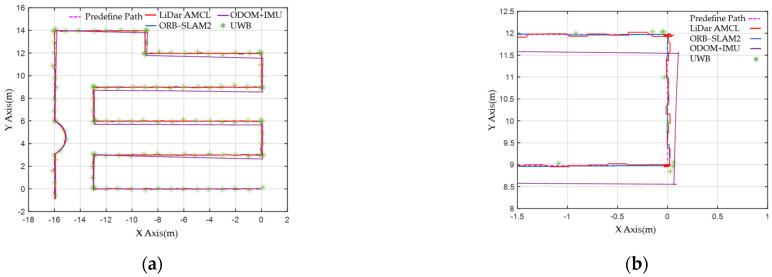
Predefined and feedback paths detected by the sensors: (**a**) The sensor results are represented by colored lines: pink dotted line: predefined path, red: LiDAR and AMCL, blue: ORB−SLAM2, purple: ODOM + IMU, green dot: UWB. (**b**) As a result of local amplification, the offset corresponding to each sensor can be found, which ODOM + IMU has a large error.

The robot system initially operates in manual control mode, recording various sensors’ detection results under obstacle-free conditions. ODOM + IMU calculates the encoder feedback at a frequency of approximately 100 Hz, as shown in Figure 13a. Due to environmental influences, cumulative errors occur, causing the deviation from the target path to increase over time. The UWB signal operates at 1 Hz, with a deviation of approximately 30 cm. The LiDAR and depth camera utilize AMCL and ORB-SLAM2 for pose estimation calculations, respectively. AMCL runs at approximately 50 Hz, while ORB-SLAM2 runs at approximately 20 Hz. Based on Figure 13b, both methods yield similar localization results, with a deviation of less than 5 cm.

#### 4.2.2. The 2-Level EKF Experiment Result

**Figure 14:**
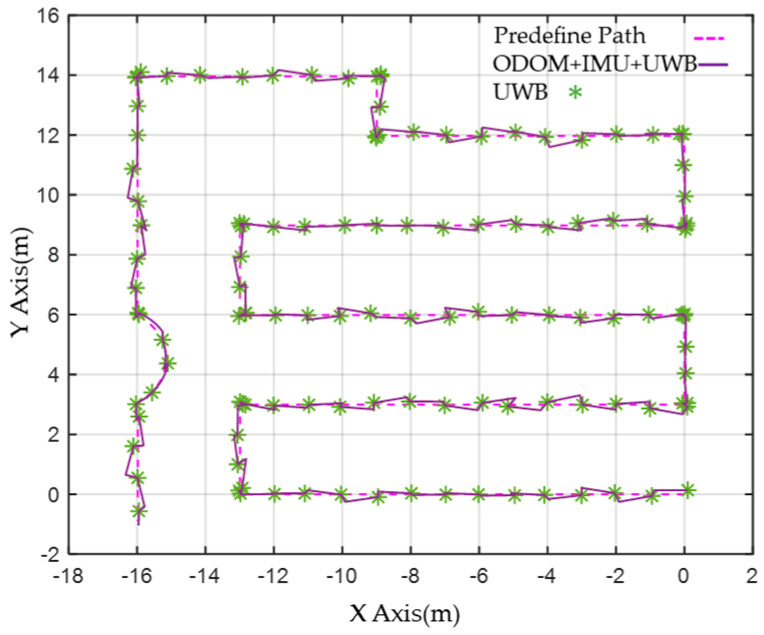
The comparison between the actual path taken in the field and the navigation path diagram produced by the level 1 EKF with UWB localization.

To address encoder error accumulation and improve drift performance, the ODOM, IMU pose, and UWB signals are combined and processed using level 1 EKF. Figure 14 shows the resulting output. Although the robot’s path may not fully align with the commanded path, the accumulated drift over time is corrected by incorporating the UWB localization signal, resulting in improved encoder signal performance.

Table 1 shows the sensor fusion settings used at each level, as described in Section 3. The level 1 EKF utilizes sensors such as an odometer, IMU, and UWB to calculate mixed feedback signals. Applying the method described above, the level 2 EKF primarily relies on LiDAR localization information (Pose A) as input. Depending on the energy-saving selector, it decides whether to perform mixed calculations with the depth camera (Pose B) or the level 1 EKF calculation result (Pose C).

**Figure 15:**
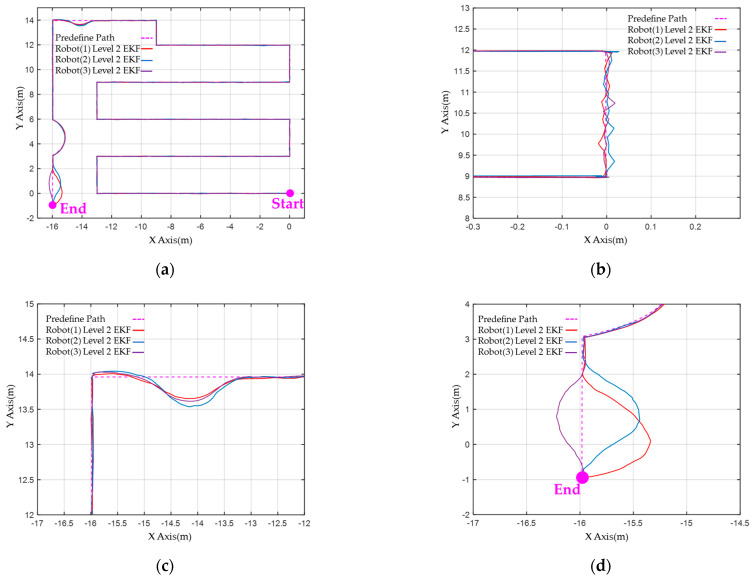
Pre-defined path and 2−level EKF calculation results in robots: (**a**) Calculation results of global planning path and 2−level EKF localization in three robots. (**b**) The 2−level EKF localization calculation results of partially enlarged scenario II. (**c**) The 2−level EKF localization calculation results of partially enlarged scenario III. (**d**) The 2−level EKF localization calculation results of partially enlarged scenario IV.

Figure 15 compares the localization performance of three robots during global navigation operations in an experimental field using a 2-level EKF with a predefined path. Figure 15a illustrates the result of a global path calculation. By employing the 2-level EKF, it was determined that the robot could navigate a vast field space using level 1 ODOM + IMU and UWB localization processes, despite experiencing drift deviation. Furthermore, the MRS operated smoothly in a large-scale complex field with 2D LiDAR and visual ORB-SLAM2.

The local path localization results of three robots in scenario II are illustrated in Figure 15b. When obstacles in the surrounding environment do not interfere, the 2-level EKF method can effectively track the predetermined path with high accuracy. Figure 15c,d demonstrates the robot’s localization outcomes while following the trajectory path in scenarios III and IV, respectively. Despite dynamic obstacles, robots avoid them, resulting in successful navigation. Although there are many interference factors in the operating field, such as fixed obstacles and light source reflections in scenario III and crowds in scenario IV, the 2-level EKF with VSLAM assistance can perform more accurate localization calculations.

**Figure 16:**
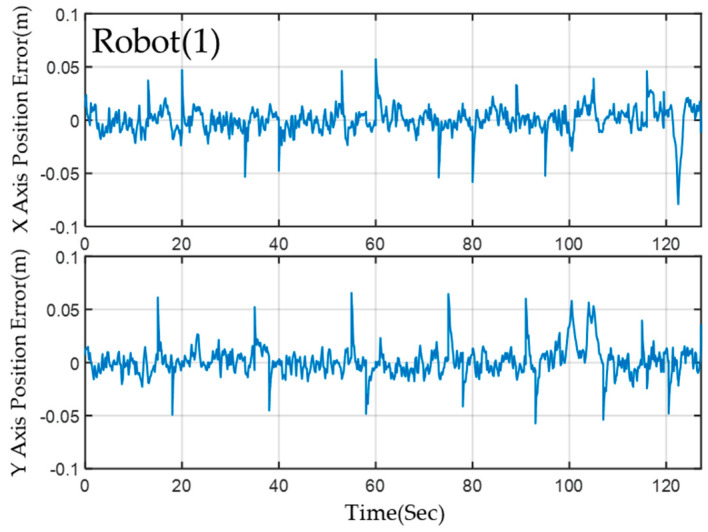
The localization error of the Robot(1) on the *x*−axis and the *y*−axis.

Despite the complex environmental variations present in the field, the MRS and robots maintain stable navigation along a predefined path owing to the successful implementation of a 2-level EKF localization calculation. This method consistently ensures accurate localization. The results demonstrate that the *X*-axis and *Y*-axis localization errors of the three robots are within a range of less than 3 cm. Figure 16 displays the results of the localization error for Robot(1) utilizing ORB-SLAM2 in conjunction with EKF fusion calculation with AMCL. Although this configuration generally provides better localization accuracy, it may exhibit transient localization deviations during rotation. However, the implementation of a 2-level EKF calculation effectively minimizes error and achieves quick convergence.

**Figure 17:**
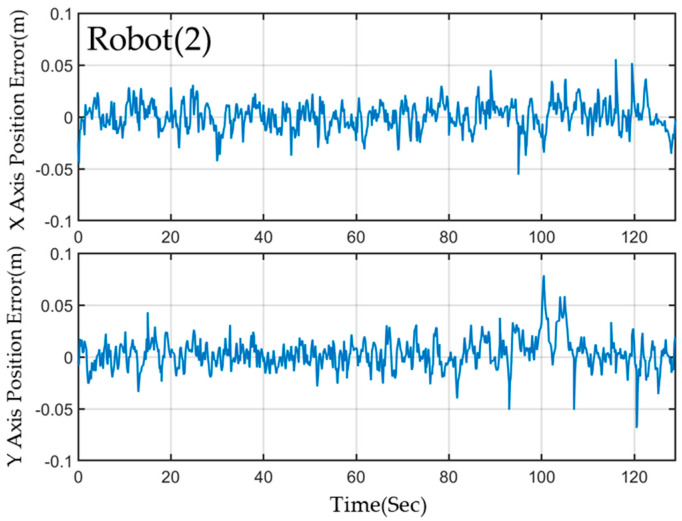
The localization error of the Robot(2) on the *x*−axis and the *y*−axis.

**Figure 18:**
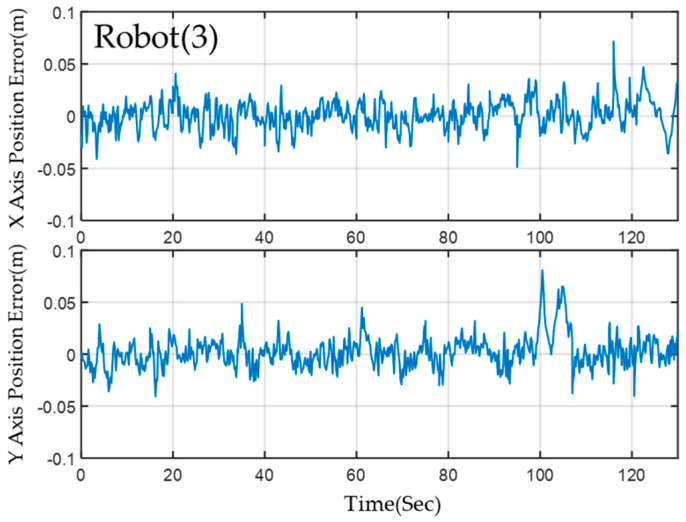
The localization error of the Robot(3) on the *x*−axis and the *y*−axis.

Figure 17 and Figure 18 present the estimation errors of motion paths for Robots (2) and (3), respectively. In scenarios I and II, ORB-SLAM2 calculation is not required, and thus, no transient localization offset occurs. However, the steady-state operating error for these robots is slightly higher than that of Robot(1). Activation of the visual localization system was necessary to facilitate rotation and obstacle avoidance upon entering scenarios III and IV. However, this led to a significant increase in estimation errors. The leading robot, which utilized visual localization assistance throughout the experiment, achieved better localization accuracy than the other robots.

**Figure 19:**
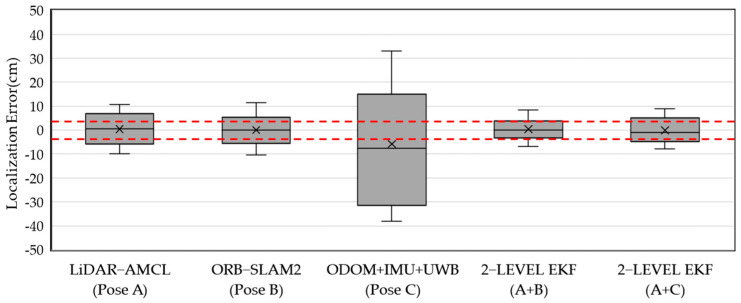
The multi-robot system runs for 100 h, and the localization error results calculated by various SLAM methods and 2−level EKF are compared.

Figure 19 presents the results of the localization error calculation analysis conducted on various sensors and the 2-level EKF during the 100 h operation of the MRS. Furthermore, this analysis compares the proposed 2-level EKF with other localization methods. LiDAR-AMCL (Pose A) and ORB-SLAM2 (Pose B) both have a primary error range of less than 10 cm, with ORB-SLAM2 having a smaller overall error but a wider deviation range. The main reason for this is that under rotational conditions, VSLAM temporarily generates larger calculation deviations. In the case of ODOM + IMU + UWB (Pose C), it is evident that errors in ODOM and IMU accumulate due to the surrounding environment, as well as the impact of UWB localization accuracy and update time. The maximum deviation can reach 30–40 cm, but error accumulation is mitigated by UWB correction, thereby preventing sustained error divergence. Comparing 2-level EKF (A+C) with Pose A, the localization error is improved by approximately 1–2 cm. On the other hand, 2-level EKF (A+B) achieves a localization error range of 3 cm, as indicated by the red dashed line. However, the error range is influenced by the ORB-SLAM2 maximum deviation and exhibits a similar widening trend. Overall, 2-level EKF (A+B) achieves the most accurate localization performance with a 3 cm error range.

### 4.3. The Energy Saving Performance Evaluation 

#### 4.3.1. The Result of the Interaction between the Energy-Saving Selector and 2-Level EKF

With the integration of a 2-level EKF localization fusion algorithm in EnergyWise MRS, service robots can navigate large and complex fields while performing disinfection tasks autonomously. By utilizing the energy-saving selector, the allocation of visual localization resources can be optimized, leading to a reduction in robot computing resources. The following is a description of the test results obtained by combining an energy-saving selector and a 2-level EKF in EnergyWise MRS, along with a discussion of the benefits of this method.

In scenario I, the leader Robot(1) of the VSLAM did not detect any obstacles, resulting in Δ*G* not exceeding *σ_i_u_*, and therefore the SW_G_ did not initiate. During acceleration and deceleration, the deviation Δ*L* between level 1 EKF localization and LiDAR localization may exceed *σ_m_u_*. In such cases, SW_L_ may temporarily replace the level 1 EKF attitude with VSLAM. Once the deviation is lower than *σ_m_L_*, the SW_L_ will switch back to the level 1 EKF pose. When entering scenario II, although there is some interference, the Δ*G* of the leader Robot(1) is insufficient to trigger SW_G_. Consequently, Robot(2) operates similarly to scenario I. In contrast, upon entering scenario III, some obstacles are too close to the leader Robot(1), causing Δ*G* to exceed *σ_i_u_*. This triggers SW_G_, and the MRS transmits the signal to Robot(2), which switches to VSLAM and level 2 EKF input signals.

**Figure 20:**
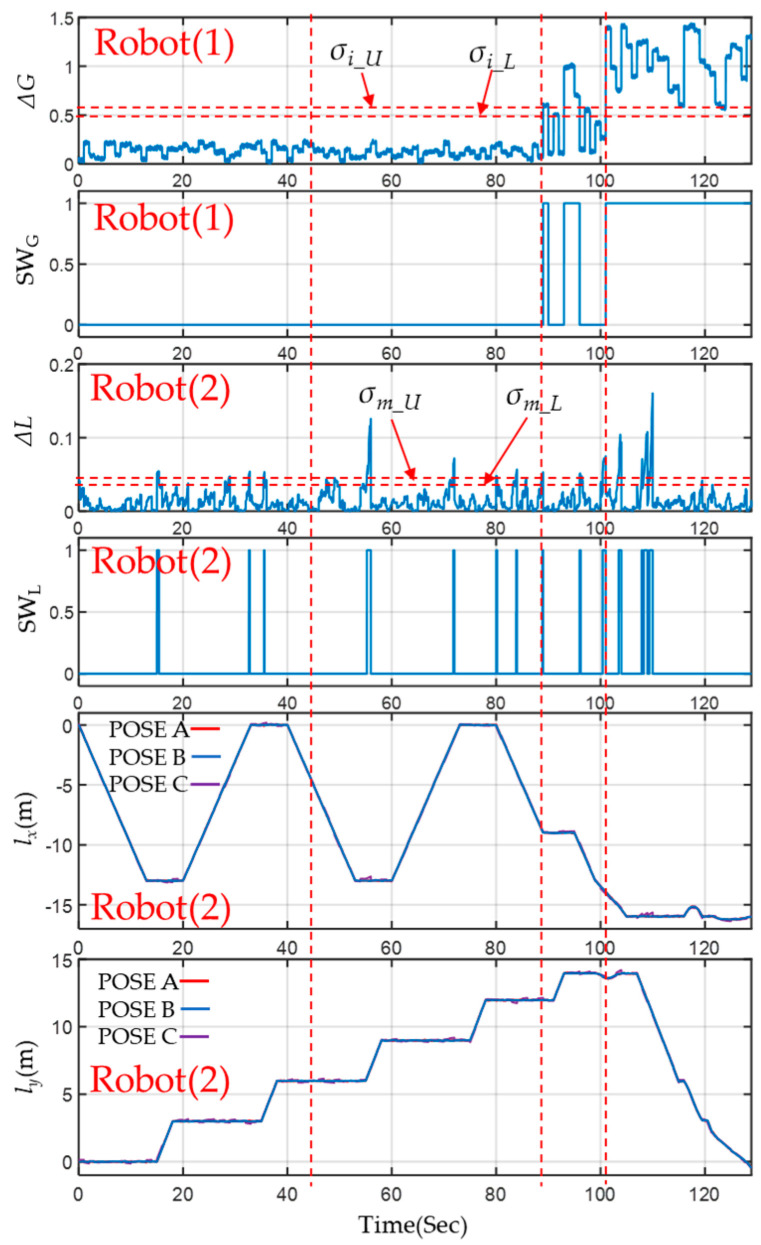
Experimental results of MRS incorporating an energy-saving selector and a 2−level EKF in each scenario.

As long as the obstacle situation persists, the Δ*G* of the leader Robot(1) remains below *σ_i_L_*, causing SW_G_ to deactivate. Therefore, Robot(2) switches off VSLAM and relies on level 1 EKF output attitude. Nonetheless, the deviation Δ*L* on Robot(2) continues to be calculated continuously, and SW_L_ is switched. In scenario IV, which involves navigating through a region with dynamic crowd obstacles, the VSLAM posture of the leader Robot(1) causes Δ*G* to consistently exceed *σ_i_u_*. As a result, the MRS keeps Robot(2) VSLAM continuously enabled to ensure that all robots can effectively position themselves and avoid obstacles. Finally, Figure 20 demonstrates that the localization calculation results of the three poses are sufficiently accurate to enable effective integration with the energy-saving selector and 2-level EKF of the MRS.

#### 4.3.2. The Result of Energy-Saving Selector

**Figure 21:**
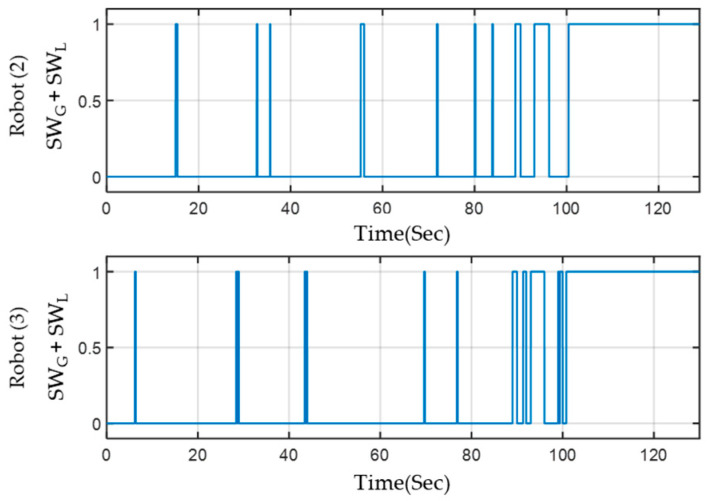
VSLAM execution time of Robots(2) and (3).

The initial waveform of the SW_G_+SW_L_ of Robot(2) and Robot(3) is shown in Figure 21. By comparing the figures above and below, we can observe that in scenarios I and II, the two robots primarily determine the opening of VSLAM based on the SW_L_ signal. In scenarios III and IV, the SW_G_ is determined by the calculation of the leader Robot(1) based on the MRS instructions. In this example, the switch waveforms of the two robots match after 89 s. Table 2 presents the relevant running time and energy-saving effect of each robot’s VSLAM calculation by organizing the start-up status of each calculation. During the experiment, the leader Robot(1) kept VSLAM turned on at all times, resulting in VSLAM consuming 3180 J of energy with no apparent energy savings observed. Robots(2) and (3) took 34.79 s and 36.06 s, respectively, to complete the VSLAM calculation, resulting in energy-saving ratios of about 73% and 72%.

Table 3 presents energy-saving results for a multi-disinfection robot system operating continuously for 10 days, including two non-working weekends. During regular weekdays, both the EnergyWise MRS and the disinfection service robot can reliably maintain approximately 125 trips per day in complex environments. Table 3 shows the 10-day accumulated operational time along with a VSLAM calculation comparison. Our analysis indicates that, compared to the previous experiment, all robots except the leading robot can benefit from the algorithmic efficiency of the energy-saving selector. Specifically, this method can reduce VSLAM computation resources by approximately 80% and improve efficiency by 54%. The results demonstrate that the energy-saving selector can significantly improve the long-term performance of the multi-robot system.

## 5. Discussion and Conclusions 

This paper developed an energy-saving-oriented multi-robot system and disinfection service robots based on ROS. The proposed EnergyWise saving selector allows the MRS to make proactive decisions on executing visual SLAM based on real-time localization information for each robot, thereby addressing the issue of reduced energy efficiency in long-term visual localization calculations. By combining EnergyWise MRS and service robots, significant energy-saving effects in VSLAM are achieved through algorithmically adjusting system resources for visual computation, resulting in an efficiency improvement of nearly 54%. The system was applied in large public environments during the COVID-19 pandemic to provide environmental disinfection services.

In addition, the integration design of UWB and 2-level EKF helps to prevent occasional localization failures of robots in large and complex environments caused by dynamic crowds and obstacles. By analyzing the recorded data of various localization methods and the outputs of the 2-level EKF during 100 h of operation in a large-scale field, it was observed that LiDAR demonstrates stable localization capability, while VSLAM achieves the highest accuracy but may experience transient localization deviations. The recorded results from the 2-level EKF indicate that the first-level EKF combined with UWB effectively mitigates the localization offset issue of ODOM + IMU, while the second-level EKF combined with 2D and VSLAM improves the overall localization accuracy to reach 3 cm.

The experimental results mentioned above confirm the effectiveness of EnergyWise MRS, the energy efficiency module, and the 2-level EKF algorithm. Therefore, the findings demonstrate that the proposed methods can be applied to service robot research. It not only enhances MRS power efficiency but also improves the localization mechanism applicable to large and complex environments. UWB can be integrated into mobile designs and assist in long-range position sensing, thus allowing research achievements to be disseminated in MRS applications to common fields.

MRS face academic challenges in information sharing, communication, and task allocation. Information sharing is limited by communication bandwidth and network topology, requiring effective communication protocols and routing algorithms. Communication can be affected by delays, failures, and interference, necessitating robust communication mechanisms. Methods to address these challenges include model predictive control, reinforcement learning, and distributed optimization. In terms of MRS and robot connections, 5G communication technology can improve multi-robot systems’ communication performance and task allocation.

Exploring the feasibility of 5G MRS is also a promising research direction. Since MRS require a large amount of data exchange and collaboration, the high bandwidth and low-latency characteristics of 5G communication technology can effectively support the information sharing and communication needs between multi-robots. In addition, 5G also provides more reliable connections and greater network capacity, which can help solve communication bottlenecks in MRS. Future research could focus on larger-scale robot control systems and facilitate diverse practical applications with faster and more reliable communication and data-sharing capabilities.

## Figures and Tables

**Table 1:** Two-level EKF sensor fusion setting table.

EKF Level	State Measurement	Configuration				
		$z_{l_{x,k}}$	$z_{l_{y,k}}$	$z_{\theta_{k}}$	$z_{v_{k}}$	$z_{{\dot{\theta }}_{k}}$
Level 2 EKF	LiDAR-AMCL (PoseA)	1	1	1	0	0
	ORB-SLAM2 (Pose B)	0	0	0	1	1
	Level 1 EKF (Pose C)	0	0	0	1	1
Level 1 EKF	Odometer	0	0	0	1	0
	IMU	0	0	0	0	1
	UWB	1	1	0	0	0

**Table 2:** The calculation and comparison of the energy-saving effects of the robots.

Robot No.	Robot Operation Time (s)	VSLAM Operation Time (s)	VSLAM Energy Consumption (J)	Energy-Saving Ratio
Robot(1)	127.2	127.2	3180	0%
Robot(2)	128.9	34.79	869.75	73%
Robot(3)	130	36.07	901.5	72.2%
MRS(Total)	386.1	198.05	4951.25	48.4%

**Table 3:** Running benefits after 10 days after importing energy-saving selector.

Robot NO.	Robot Operation Time (h)	VSLAM Operation Time (h)	VSLAM Energy Consumption (kJ)	Energy-Saving Ratio
Robot(1)	74.2	74.2	6678	0%
Robot(2)	70.6	13.6	1226	81%
Robot(3)	67.9	12.5	1124	82%
MRS(Total)	212.7	100.3	9028	54%

## Data Availability

The data presented in this study are available upon request from the corresponding author.
